# First-time detection and identification of the *Mycobacterium tuberculosis* Complex members in extrapulmonary tuberculosis clinical samples in south Tunisia by a single tube tetraplex real-time PCR assay

**DOI:** 10.1371/journal.pntd.0005572

**Published:** 2017-05-05

**Authors:** Mariam Siala, Salma Smaoui, Wafa Taktak, Salma Hachicha, Asma Ghorbel, Chema Marouane, Sana Kammoun, Dhikrayet Gamara, Leila Slim, Radhouane Gdoura, Férièle Messadi-Akrout

**Affiliations:** 1 Department of Biology, Preparatory Institute for Engineering Studies, Sfax, University of Sfax-Tunisia; 2 Department of Life Sciences, Research Laboratory of Environmental Toxicology-Microbiology and Health (LR17ES06), Faculty of Sciences, Sfax, University of Sfax-Tunisia; 3 Department of Microbiology, Regional hygiene care mycobacteriology laboratory, Hedi-Chaker University Hospital, Sfax, Tunisia; 4 Department of Biology B, Faculty of pharmacy, Monastir, University of Monastir-Tunisia; 5 Department of Microbiology, National Reference Laboratory of Mycobacteria, Research Unit (UR12SP18), A. Mami University Hospital of Pneumology, Ariana, Tunisia; 6 Care management and basic health, Ministry of Health, Tunis, Tunisia; Swiss Tropical and Public Health Institute, SWITZERLAND

## Abstract

**Introduction:**

Tunisia has one of the highest burdens of extrapulmonary tuberculosis (EPTB) among tuberculosis (TB) cases but the contribution of MTBC-mediated human EPTB is unknown. EPTB diagnosis is challenging due to the paucibacillary nature of clinical samples. Therefore, a need of a simplified molecular method for sensitive and specific TB detection and differentiation of MTBC members caused EPTB remains a priority to an early diagnosis, optimize successful anti-TB treatment and minimize transmission. We evaluated the performance of a single tube tetraplex Taq Man real time PCR for EPTB detection and differentiation between MTBC members directly on extrapulmonary samples.

**Materials and methods:**

Extrapulmonary samples obtained from clinically suspected EPTB patients from 2013 to April 2015 were tested by Ziehl Neelsen Staining, mycobacterial culture and qPCR assay for RD1, RD9, RD12 and ext-RD9 targets (MTBC-RD qPCR). The performance of qPCR was compared to a reference standard based on MTBC culture and/or at least two criteria of a composite reference standard (CRS) including clinical, radiological, histopathological and therapeutic findings.

**Results:**

EPTB was identified in 157/170 (92.4%) of included patients of whom 99 (63%) were confirmed by culture and 58 (36.9%) by CRS criteria. The sensitivity and specificity of qPCR, in comparison to the reference standard were 100% (157/157) and 92.3% (12/13), respectively. The sensitivity of qPCR was statistically significant as compared to culture and smear microscopy (*P*< 0.001). QPCR results showed *M*. *bovis* identification in 77.1% of extrapulmonary samples in occurrence to lymphadenitis infection. *M*. *tuberculosis* and *M*.*bovis BCG* were detected in 21.6% and 1.3% of cases, respectively.

**Conclusions:**

MTBC–RD qPCR proved to be a rapid and sensitive assay for simultaneously TB detection and MTBC members identification on extrapulmonary samples within 1.5 days after sample receipt. Its high sensitivity could make this method a useful tool in diagnosing TB in addition to routine conventional methods and TB clinical parameters.

## Author summary

Mycobacterial related EPTB diagnosis remains a challenge. In fact the paucibacillary nature of human specimens realized from inaccessible sites might be one of the causes giving a low sensitivity of routine used diagnostic tests. Therefore the use of Real time PCR (qPCR) contributes to a specific, sensitive and rapid EPTB diagnosis which helps to a successful anti TB treatment. However almost all the previous studies using qPCR to improve the molecular diagnosis of EPTB have focused only on positive culture materials or have identified only the *Mycobacterium* genus. Indeed, despite the relatively high occurrence of TB cases in Tunisia, there is no study available on EPTB related mycobacteria in southern Tunisia. Thus, our study is the first to evaluate a single tube tetraplex MTBC-RD qPCR in order to (i) detect and differentiate between the different MTBC members directly on EPTB specimens (ii) correlate qPCR results with a reference standard based on culture and/or at least two criteria of a composite reference standard (CRS) including clinical, radiological, histopathological and therapeutic findings. In our study, MTBC-RD qPCR was shown to give a high sensitivity and specificity compared to the reference standard. *M*. *bovis* is the major cause of EPTB in occurrence to lymphadenitis infection. Finally, *M*. *bovis* and *M*. *tuberculosis* were identified by qPCR among patients with negative culture being CRS positive for EPTB.

## Introduction

Tuberculosis (TB) remains a leading cause of morbidity and mortality worldwide [1]. According to annual surveys conducted by the World Health Organization (WHO), 10.4 million new active TB cases and 1.8 million deaths occurred in 2015 [1]. Although pulmonary TB is the most common presentation of this disease, it can involve any organ in the body [2]. Extrapulmonary Tuberculosis (EPTB) is defined as the isolated occurrence of TB in any part of the body other than lungs [2]. The prevalence of EPTB is highly variable which is essentially attributable to the geographic origin of the patient. A high incidence is observed among the immunocompromised HIV co-infected patients [3]. In Tunisia, EPTB makes up to 57% of TB cases despite the low prevalence of HIV which is higher compared to other countries [4]. EPTB can be caused by *Mycobacterium tuberculosis* complex (MTBC), such as *Mycobacterium tuberculosis*, *Mycobacterium bovis*, *Mycobacterium bovis* BCG and *Mycobacterium africanum* [5]. An early and rapid TB diagnosis as well as distinction between the different MTBC members are essential to determine the EPTB etiology and to optimize efficient anti-TB treatment since *Mycobacterium bovis* and *Mycobacterium bovis* BCG are intrinsically resistant to pyrazinamid (PZA), an important first-line anti-TB drug [6, 7]. Indeed, the natural mode of infection and surveillance measures for EPTB differ between complex members. For example, early diagnosis of *M*. *bovis* might prompt questions to ascertain the risk factors of zoonotic exposure or a contamination of derived food/dairy products from diseased cattle, the primary routes of EPTB infection [2, 5].

In fact, the diagnosis of EPTB poses difficulties due to the diverse manifestations which may mimic other pathologies [8, 9], the difficulty of sampling, the paucibacillary nature of specimens as demonstrated by the low sensitivity of acid-fast bacilli smear as well as culture methods and a longer incubation delay for the growth of mycobacteria [5, 8, 9]. Indeed, the most commercially molecular tested assays are of limited use in differentiation between MTBC species and which were not evaluated directly on clinical specimens. Therefore, there is a great need of molecular simplified amplification methods for rapid, sensitive and specific detection and differentiation of MTBC members directly on clinical specimens. The new generation of real-time PCR (qPCR) has been particularly developed for these purposes [6, 10]. Recently, comparative genomics of the MTBC identified several regions (regions of difference; RD), ranging in size from 2 to 12.7 Kb, that were present in *Mycobacterium tuberculosis* H37Rv and absent in others members of MTBC. These results suggested that deletion of genomic regions have contributed to generating genetic diversity within this complex [11]. Based on these findings, Halse *et al*, have published a single-tube, multiplex protocol of Taq Man qPCR assay (MTBC-RD qPCR) developed for rapid detection and differentiation of MTBC members from TB clinical specimens. This MTBC-RD qPCR was based on comparative genomic deletion analysis, using RD motifs that are either common to MTBC members or specific to each one [6]. To the best of our knowledge, no studies concerning EPTB-related infectious mycobacteria in south Tunisia have previously been conducted. Accordingly, in this study, we evaluated the performance of a single tube tetraplex MTBC-RD qPCR for EPTB detection and differentiation between MTBC species from extrapulmonary samples.

## Materials and methods

### Study patients and sample processing

In our study, non redundant specimens were obtained from patients with suspected EPTB infection in any extrapulmonary site from January 2013 to April 2015. All analyses were performed prospectively in the regional hygiene care department of Microbiology, Mycobacteriology Laboratory of Hedi-Chaker University Hospital in Sfax (South of Tunisia). The laboratory provides routine mycobacterial diagnostic tests for TB specimens obtained during clinical routine diagnosis for consulting or hospitalized suspected TB patients in Hedi-Chaker University Hospital.

Patients (adults and children, i.e. individuals< 18 years of age) with suspected EPTB based on clinical signs and symptoms, cytological /histological and/or radiological signs suggesting TB were eligible for our study. Patients were excluded in case of they had a contaminated culture; when they were treated with an anti-TB treatment (ATT) within the past 1 month from the first presentation to the clinician and if they died.

Specimens were aliquoted upon arrival in the laboratory. One aliquot was processed in the mycobacteriology laboratory for Ziehl Neelsen staining (ZNS) technique, culture and identification of mycobacteria. Another aliquot was used freshly or after storage at -80°C for DNA extraction and MTBC-RD qPCR performed in the research laboratory separately by another technician anonymously to clinical results. Approval for usage of the remaining clinical specimens for our study was obtained by the Ethics Committee of Hedi Chaker Hospital.

#### Smear microscopy

The ZNS method for the presence of acid fast bacilli (AFB) was performed as described previously [12].

#### Culture and identification of mycobacteria

The specimens obtained after liquefaction and decontamination using the N-acetyl-L-cysteine, sodium hydroxide (NaOH-NALC) were cultured in both solid (Lowenstein-Jensen (LJ) and Coletsos) and liquid (MGIT 960; Becton Dickinson Biosciences, Sparks, MD, USA) media. Identification of mycobacterial isolates was based on morphological characteristics of colony (growth time, colony form and pigment production) and the results of standard biochemical tests [13]. These latter included niacin test, nitrate reductase test, and susceptibility for certain inhibitors such as thiophene-2-carboxylic acid hydrazide (TCH) and pyrazinamid and growth on p-nitrobenzoic (PNB) medium. To confirm morphological and biochemical identification, all isolates were identified by the GenoType MTBC test (Hain Lifescience GmbH, Nehren, Germany) according to the instructions of the manufacturer [14].

### DNA extraction

For DNA extraction, 50 mg of the decontaminated and grinded specimen was heat treated at 80°C for 1 h before using the High Pure PCR Template Preparation kits (Roche, Rotkreuz, Switzerland) as described by the manufacturer. An additional lysis treatment step using lysozyme was included in the protocol following the proteinase k digestion. Briefly, the suspension was resuspended in 200 μl of lysis buffer and 40 μl of proteinase k. The mixture was incubated immediately at 56°C overnight. 200 μl of supernatant obtained after centrifugation at 5000 *g* for 5 min, were mixed with 5 μl lysozyme solution followed by incubation in thermo mixer (Eppendorf) at 37°C and 550 rpm for 15 min. Then, 200μl of binding buffer was added. The sample was incubated immediately at 70°C for 10 min. The spined solution was added to the DNA binding columns provided by High Pure PCR Template Preparation kit and processed as described by the manufacturer. Finally, the DNA template was eluted in 100 μl of the elution buffer and used as template in PCR-protocol. DNA extraction was performed in a biological hood using filter tips (Tip One; Starlab, Bagneux, France) and gloves were changed between each sample.

### MTBC-RD real-time PCR details

A multiplex protocol of Taq Man real-time PCR for the detection and differentiation between the MTBC members was performed to test the DNA samples extracted from EPTB human specimens, as well as positive and negative controls, following a protocol previously described by Halse *et al* [6]. We have used the same primers and probes of the original protocol for RD1, RD9, RD12 and ext-RD9 targets without the use of RD4 one since they are sufficient to distinguish between MTBC members as demonstrated in Table 1 [6]. Briefly, the qPCR was performed in a 25 μl final volume with Ex Taq Premix Tli RNaseH Plus (Takara, Japan) as described previously [6]. QPCR was performed on a CFX96TM real-time PCR cycler (Biorad, USA). Pure DNA was amplified in duplicate without and with a 1:5 dilution. Thorough preventive measures were taken to avoid DNA contamination during extraction and qPCR manipulation as mentioned previously [15].

**Table 1:** QPCR positivity and negativity based on molecular specific RD^1^ patterns for each species of MTBC.

Mycobacteria	RD qPCR result			
	RD1	RD9	RD12	ExtRD9
***M*.*tuberculosis***	+	+	+	+
***M*. *bovis***	+	-	-	+
***M*. *bovis BCG***	-	-	-	+
***M*. *africanum***	+	*-*	+	+
***M*. *microti***	-	-	+	+
***M*. *canettii***	+	+	-	+
**NTM2**	-	-	-	-

^1^RD: regions of difference, NTM^2^: nontuberculous mycobacteria

### Case definition

The reference standard for EPTB diagnosis was defined as a positive culture for MTBC and/or a positivity of at least two criteria of a composite reference standard (CRS) including: (a) TB clinical symptoms, (b) histology/cytology findings, (c) radiological tests (site specific computerized tomography scan/ magnetic resonance imaging), (d) therapeutic response to ATT.

The histology/cytology findings of the specimen were defined positive by the presence of caseation necrosis and/or epithelioid granuloma. Radiological positive tests were noted when infiltrates or cavities, hilar lymph nodes, pleural effusions, or tuberculomas, leptomeningeal and basal cistern enhancement were found. A positive response to ATT therapy was considered when patient had clinically improvement 3 months after the date of enrollment.

Diagnosis and clinical management of patients were done according to the reference standard and to clinician report.

### Data analysis

Analyses were done using Epi Info (Info 7) and SPSS 13.0 (SPSS; Chicago IL) softwares. Sensitivity, specificity, positive and negative predictive values with 95% confidence intervals were calculated for MTBCRD-qPCR accuracy, against MTBC culture and against the reference standard based on culture and/or CRS criteria according to the Standards for Reporting of Diagnostic Accuracy Studies (STARD) recommendations [16].

## Results

### Patients study

**Fig 1:**
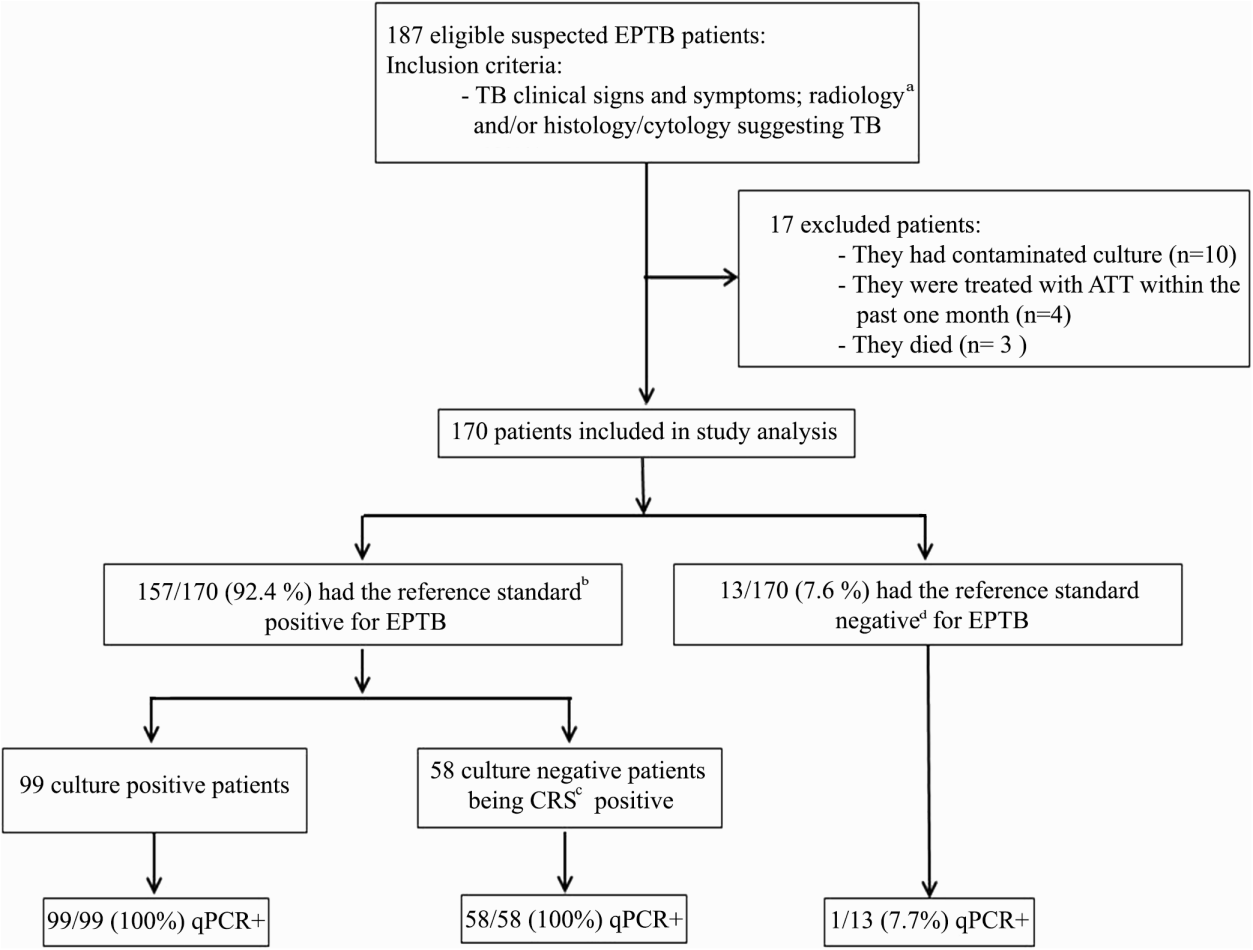
Study flowchart. ^**a**^CT: computerized tomography scan, MRI: magnetic resonance imaging; ^**b**^ Reference standard = MTBC culture + and /or at least two + CRS criteria; ^**c**^CRS: Composite reference standard: (i) TB clinical symptoms, (ii) histology/cytology findings, (iii) radiological tests, (iv) therapeutic response to anti-TB treatment (ATT). ^**d**^culture and CRS were negative and patient had improvement without ATT. +: positive.

Of the 187 eligible patients, 17 were excluded. Thus, 170 patients were included in the study analyses (Fig 1 for flow chart study details).

Available specimens were 144 lymph nodes biopsy samples (fine needle aspirates, n = 114, and tissues, n = 30), as well as 26 additional samples including pus and abscess (n = 10), 9 body fluids including cerebrospinal fluid (CSF) (n = 8) and pleural fluid (n = 1), and finally 5 tissue and 2 bone scarping samples.

The age range of our patients was 4 months to 80 years with a mean age of 31.3 years (SD ± 16.93 years). The majority of cases 78.8% (134/170) were adults and 21.2% (36/170) were children (aged <18 years). The sex-ratio was 0.54. The majority of patients 71.2% (121/170) originated from southern governorates of Tunisia and 28.8% (49/170) from Sfax. Contact with livestock farming or cattle was identified in 23 cases and ingestion of unpasteurised dairy products and milk in 53 cases. In addition, 3 patients had a possible risk of exposure related to their family contact and 91 patients had no information records. None of the patients was tested positive for HIV antibodies.

Out of the 170 patients, 99 (58.2%) had positive cultures for MTBC on MGIT 960 liquid and/or solid media (Table 2); 58 (34.1%) culture-negative patients had EPTB diagnosis based on CRS which were clinically, radiologically, and/or histologically/cytologically positive (Table 2). All patients were positive for ATT response after follow up (Table 2). Smear microscopy was positive for AFBs in 29/170 specimens (17.1%) from patients of whom 17 had positive MTBC culture and 12 being CRS positive for EPTB. Thirteen out of 170 patients (7.6%) who had the reference standard negative for EPTB (Table 2), had other non-TB diagnoses including: viral meningitis (n = 2), sarcoidosis (n = 1), brucellosis (n = 3), aspergillosis (n = 2); Non Hodgkin lymphoma (n = 3), thyroid abscess (n = 1) and mycobacteriosis (n = 1). Thus, in total, EPTB was identified in 157 /170 (92.4%) of the included patients (Fig 1).

**Table 2:** Reference standard details for EPTB diagnosis.

EPTB definition	Reference standard (Culture and /or CRS)				
	MTBC Culture	Clinical symptoms	Radiology^a^	Histology/cytology^b^	Follow-up after 3months^c^
**(n = 99)**	+	+	+/-	+/-	+
**(n = 42)**	-	+	+	+	+
**(n = 8)**	-	+		+	+
**(n = 8)**	-	+	+		+
**NotTB**^d^ **(n = 13)**	-	+	-	-	NT^e^

^**a**^: Radiological positive tests were noted when infiltrates or cavities, hilar lymph nodes, pleural effusions, or tuberculomas leptomeningeal and basal cistern enhancement were found.

^**b**^: The histology/cytology findings of the specimen were positive with the presence of caseation necrosis and/or epithelioid granuloma.

^**c**^: A positive response was considered if patient had received ATT and had improvement after 3 months from the date of enrollment.

^d:^ Culture and CRS were negative and patient had improvement without ATT

^e^NT: not treated with anti-TB drugs.

EPTB samples were culture positive for *M*. *bovis* (76/99, 76.8%), *M*. *tuberculosis* (21/99, 21.2%) and for *M*. *bovis* BCG (2/99, 2%). One NTM strain was isolated. Thus, 99 specimens were considered as true positive for MTBC growth.

### Accuracy of MTBC RD qPCR, smear microscopy and culture for EPTB diagnosis

The sensitivity of smear microscopy and culture was 18.5% (29/157) and 63% (99/157), respectively among patients with a positive reference standard (Table 3). MTBC RD qPCR was positive in 157/157 (100%) of patients with EPTB diagnosis considered as true positive based on the reference standard and in one non TB patient 1/13 (7.7%) demonstrating one false positive case.

**Table 3:** Accuracy of MTBC RD qPCR assay, smear microscopy and culture.

	Sensitivity			Specificity			PPV		NPV	
	n/N	(%)	95% CI	n/N	(%)	95% CI	(%)	95% CI	(%)	95% CI
**Smear microscopy vs RS**^a^	29/157	(18.5)	12.9–25.6	12/12	(100)	71.6–100	100	85.4–100	9.2	5–15
**Culture vs RS**	99/157	(63)	55–70.5	13/13	(100)	71.7–100	100	95.3–100	18.31	10.5–29.6
**RD qPCR vs RS**	157/157	(100)	97–100	12/13	(92.3)	62.1–99.6	99.4	96–100	100	69.9–100
**RD qPCR vs Culture**	99/99	(100)	95.3–100	12/71	(16.9)	9.4–28.1	62.7	54.6–70.1	100	69.9–100

^a^RS: reference standard for EPTB is defined as positive culture and/or at least two positive CRS* criteria; *CRS: Composite reference standard: (i) TB clinical symptoms, (ii) histology/cytology findings, (iii) radiological tests, (iv) therapeutic response to anti-TB treatment (ATT); PPV: positive predictive Value; NPV: Negative Predictive Value; n: index group; N: control group; CI: confidence interval

Upon comparison with the reference standard, the sensitivity of the MTBC-RD qPCR was 100% (157/157) which is statistically significant as compared to culture (*P*< 0.001) and smear microscopy findings (*P*< 0.001). The specificity of AFB smear, culture and MTBC-RD qPCR tests was 100%, 100%, 92.3%, respectively. The overall sensitivity of the MTBC-RD qPCR test compared to culture was 100% (99/99) with a lower specificity of 16.9% (12/71). Table 3 presents the sensitivity, specificity positive and negative predictive values with 95% confidence intervals of MTBC RD qPCR assay, smear microscopy and culture.

### MTBC species identification

*M*. *bovis*, *M*. *tuberculosis*, and *M*. *bovis BCG* were identified in 77.1% (121/157), 21.6% (34/157) and 1.3% (2/157) of extrapulmonary samples, respectively by MTBC-RD qPCR (Table 4). *M*. *tuberculosis* was identified in one out the 13 specimens from non TB patients.

*M*. *bovis* and *M*. *tuberculosis* infection were identified in 45 (28.7%) and 13 (8.3%), respectively of culture negative specimens from patients being CRS positive for EPTB. Regardless of the main site of EPTB infection, *M*. *bovis* was present exclusively in 80.4% of cases from the lymphatic population (111/138) compared to 19.6% (27/138) for *M*. *tuberculosis*. However, *M*. *bovis* and *M*. *tuberculosis* were detected with different proportion in cases with non lymphadenitis infection (52.6% vs 36.8%), respectively. *M*. *bovis* BCG was detected in pus of 2 children post BCG vaccination (Table 4).

**Table 4:** MTBC species identification by multiplex MTBC-RD qPCR on extrapulmonary specimens in EPTB patients according to the reference standard*.

Clinical specimens	MTBC species identification by multiplex MTBC-RD qPCR					
	*M*. *bovis*, n = 121 (77.1%)		*M*. *tuberculosis*, n = 34 (21.6%)		*M*. *bovis* BCG, n = 2 (1.3%)	
	Culture pos ± AFB	Culture neg CRS** pos	Culture pos ±AFB	Culture neg CRS pos	Culture pos ±AFB	Culture neg CRS pos
**Lymph nodes**^a^ **(n = 138)**	73	38	15	12	0	0
**Pus and** abscess **(n = 8)**	0	2	4	0	2*	0
**Body fluid**^b^**(n = 7)**	1	5	0	1	0	0
**Tissue**^c^ **(n = 3)**	2	0	1	0	0	0
**Bone scrapings (n = 1)**	0	0	1	0	0	0
**Total (n = 157)**	76 (48.4%)	45 (28.7%)	21 (13.4%)	13 (8.3%)	2^d^ (1.3%)	0

MTBC: *Mycobacterium tuberculosis* complex

*reference standard for EPTB is defined as positive culture and/or at least two positive CRS** criteria

**CRS: Composite reference standard: (i) TB clinical symptoms, (ii) histology/cytology findings, (iii) radiological tests, (iv) therapeutic response to anti-TB treatment (ATT); AFB: Acid Fast Bacilli; pos: positive, neg: negative

^**a**^: Lymph nodes included biopsies (n = 30) and fine needle aspiration (n = 114).

^**b**^: Body fluid included CSF (n = 8) and pleural fluid (n = 1).

^**c**^: Specimen source comprised muscle, bronche, pleural and synovial tissue.

^d^: Patients post BCG vaccine.

Among all samples tested by MTBC-RD qPCR, 99 had paired MGIT liquid and/or solid media culture with biochemical and Genotype MTBC tests species identification results for comparison. In total, 97 specimens (97.9%) were concordant by qPCR and the latter methods and 2 were discordant (1 *M*. *bovis* and 1 *M*. *tuberculosis*). The identification showed the occurence of MTBC as follows: *M*. *tuberculosis* (20), *M*. *bovis* (75), *M*. *bovis* BCG (2) (Table **5**). The concordance of *M*. *tuberculosis*, *M*. *bovis* and *M*. *bovis* BCG was 95.2% (20/21) and 98.7% (75/76) and 100% (2/2) respectively (Table **5**).

**Table 5:** Comparison of the qPCR results to the other conventional methods^a^ for MTBC species identification.

Identification with MTBC–RD qPCR	Results with conventional identification^a^				Total
	*M*. *tuberculosis*	*M*. *bovis*	*M*. *bovis BCG*	Negative	
*** M*. *tuberculosis***	20	1		13	34
*** M*. *bovis***	1	75		45	121
*** M*. *bovis BCG***			2		2
**Negative**				0	0
**Total**	**21**	**76**	**2**	**58**	**157**

^**a**^: Conventional identification is based on morphological, biochemical and molecular identification with GenoType MTBC test.

## Discussion

In this study, a single tube tetraplex MTBC-RD qPCR assay for the simultaneous detection and identification of MTBC species directly on extrapulmonary specimens was evaluated and was compared to the conventional methods. Here, we have chosen a qPCR test based on the amplification of specific mycobacterial RD motifs. Their presence or absence indicates a specific molecular profile that could differentiate between different MTBC species [6, 10, 17]. Though the identification of MTBC members based on the detection of RD patterns by PCR has been suggested previously [10], the majority of the published data used syber green detection and melting curve or conventional PCR or focused solely on positive culture materials [10, 18, 19, 20, 21]. There is only one study which evaluated the MTBC-RD qPCR directly on clinical specimens [6]. However, this latter work relied essentially on TB specimens initially positive for MTBC by IS6110 qPCR of which 5.7% were extrapulmonary samples compared to 94.3% from pulmonary TB which can be diagnosed more easily than EPTB [6]. Thus, our current study is the first to evaluate the MTBC-RD qPCR for the presence of MTBC DNA directly on 170 clinical non-respiratory specimens from patients with suspected EPTB. Thus, we could demonstrate that MTBC RD qPCR detected MTBC DNA in 100% (99/99) of samples from patients that were microbiologically confirmed EPTB using MTBC culture. MTBC RD qPCR was positive also in 58/58 (100%) of specimens from patients with EPTB diagnosis based on the CRS criteria. Upon comparison with the reference standard, the sensitivity of the MTBC-RD qPCR was 100% (157/157) which is statistically significant compared to culture (99/157) (*p* = <0.001) and smear microscopy (29/157) (*p* = <0.001). Twelve out of 13 specimens from patients who had a reference standard negative for EPTB (true negative patients) were negative by MTBC-RD qPCR indicating a specificity of 92.3% of this molecular assay. When comparing qPCR accuracy to culture known as the basic gold standard, the specificity was much lower (16.9%, 12/71) due to the lower sensitivity of the culture. Thus, our findings emphasize that the MTBC- culture most likely underestimates the mycobacterial detection i.e the diagnosis of EPTB. This could be essentially due to the paucibacillary nature of the extrapulmonary specimens and especially those from childhood TB or to the presence of other microorganisms in the same culture that have overgrown MTBC [22, 23, 24, 25, 26]. As reported previously, EPTB diagnosis requires an elaborated diagnostic algorithm based on the use of molecular methods such qPCR (e.g. GeneXpert) which is critically dependent on the CRS based on clinical diagnosis TB parameters [25, 26]. Accordingly, our data also showed and extend previous studies that the use of qPCR on non respiratory materials could be the method of choice for a rapid, specific and sensitive EPTB detection. Indeed, the current study raises the issue of the reference standard based on culture and/or CRS criteria to be used in the comparative evaluation of our tetra-plex qPCR test rather than considering only culture positivity or conventional PCR as a basic standard for EPTB detection. Therefore, its high sensitivity, reliability and ease of use could make this method a useful tool in diagnosing TB in addition to routine conventional diagnostic tests and TB clinical parameters. The high sample size of extra-pulmonary lymphatic specimens used in our study could be an additional advantage to evaluate the post decision of the clinical utility of this assay in EPTB clinical settings. The MTBC-RD qPCR seems to be useful regardless of the specimen type especially in lymph node biopsies and aspirates which constituted 84.7% of all samples. However, the low sample size of analyzed specimens from cases without lymphadenitis infection is the weak point of this work.

In addition, the strength of our approach, i.e. the high qPCR sensitivity most likely results from the combination of four different Mycobacterial targets in one reaction step, the stringent precautionary measures taken at each step to prevent a possible contamination as well as the use of an appropriate DNA preparation method. It has also been chosen to maximize extraction efficiency by minimizing the potentially inhibiting effect of extra pulmonary samples inhibitors on the *taq* polymerase. A maximum sensitivity is essential when the main objective is the amplification of a potentially low bacterial copy number such as EPTB related *Mycobacterium* thus allowing a reliable diagnosis and a rapid initiation of an appropriate drug treatment [6, 27].

In the present study, multiplex MTBC-RD qPCR targeting four different mycobacterial genes enabled the specific identification of *M*. *bovis*, *M*. *tuberculosis* and *M*. *Bovis* BCG DNA in 77.1%, 21.6% and 1.3% of clinical extrapulmonary specimens from all EPTB patients. We found a good concordance (97.9%) between qPCR and conventional methods which relied on a well validated procedure based on the combination of culture, biochemical and Genotype MTBC tests used only on isolated strains to differentiate the MTBC species. In fact, it would be worth using the combination of these three assays for mycobacterial identification. However, the limitations of culturing are still unavoidable (time delay of 4–12 weeks for mycobacterial isolation); in addition, biochemical assays are slow, cumbersome, imprecise and non-reproducible [17, 18, 28]. Indeed, the Genotype MTBC assay involves several separate reactions requiring post amplification steps thus increasing the contamination risk and/or the delay of mycobacterial identification. Consequently, the MTBC RD qPCR is significantly sensitive and able to provide a rapid identification yielding a diagnosis within 1.5 days after sample receipt.

*M*. *bovis* and *M*. *tuberculosis* DNA were detected in 59 culture-negative samples which were not included in the comparative study. Indeed, there were 2 culture-positive samples showing discordant MTBC species identification (1 *M*. *bovis* and 1 *M*. *tuberculosis*) that were misidentified by MTBC-RD qPCR. They were further tested in a singleplex test using only the RD9 target and one presented a positive amplification result and the other one provided a negative RD9 signal. Our findings were concordant with those of Halse *et al* who reported a highly specific and sensitive multiplex qPCR for detection of 376 *M*. *tuberculosis*, 15 *M*. *bovis*, 12 *M*. *africanum* and 6 *M*. *bovis* BCG but misidentifications were found in 2 culture-positive *M*. *tuberculosis* samples misidentified as *M*. *africanum* showing a negative RD9 signal amplification [6]. The authors explained this misidentification by the fact that the RD9 set of primers and probe is less efficient than the other used RD target [6]. Therefore, it is possible that the complexity of the single tube tetra-plex qPCR associated with the composition of one particular sample could lead to false-negative results for RD9 target.

In Tunisia, so far there is no molecular study available on EPTB related infectious myco-bacterial species even though this country is known to have a relatively a high prevalence of TB cases [4]. Interestingly, Ghariani *et al* have shown a high prevalence of *M*. *bovis* (76%) as a causative agent of lymphadenitis TB in north Tunisia evaluating only culture positive lymph node specimens by classical conventional methods [29]. In south Tunisia, the contribution of *M*. *bovis*-mediated human EPTB is unknown. Multiplex MTBC-RD qPCR results found in the current study extend those reported by Ghariani *et al* and could also demonstrate *M*. *bovis* identification in 77.1% of extrapulmonary samples in occurrence to lymphadenitis infection. Indeed, *M*. *tuberculosis* was detected in 21.6% of cases. However, *M*. *bovis*, the agent of bovine TB, may still be considered a potential cause of human cases, especially in developing countries where control measures for bovine TB in cattle and/or milk and dairy products are not always satisfying [3]. However, our findings emphasize that EPTB *M*. *bovis* disease is very likely underestimated in Tunisia since the control measures for herd, livestock and unpasteurized dairy products as well as milk ingestion are consistently declining. In fact, small cattle herds were dominant in the private sector representing 70% of cattle livestock posing many challenges for control and prevention to veterinary medicine [30]. The consumption of unpasteurized milk and dairy products is still traditionally widespread among many people especially in rural areas. Of note, most of our patients 71.2% (121/170) originated from southern governorates of Tunisia, a rural area with many animal breeding centers. From a clinical perspective, the genetic characterization of the *M*. *bovis* population implicated in human EPTB using spoligotyping and MIRU-VNTR is of considerable interest in order to confirm not only the species identification but also yields further insights in the diversity and dynamics of *M*. *bovis* strains circulating in this particular setting.

In conclusion, we present data of a single tube tetra-plex qPCR assay for the detection and differentiation of MTBC species. As a result of this thorough evaluation, MTBC–RD qPCR proved to be a rapid and sensitive assay for simultaneously detecting TB and differentiating MTBC members on extrapulmonary samples. This diagnostic approach contributes valuable and reliable information allowing an optimal therapeutic regimen and helping to avoid further TB transmission.
